# Food Patterns are Associated with Likelihood of CKD in US Adults

**DOI:** 10.1038/s41598-018-27365-6

**Published:** 2018-07-16

**Authors:** Mohsen Mazidi, Hong-kai Gao, Andre Pascal Kengne

**Affiliations:** 1 Department of Biology and Biological Engineering, Food and Nutrition Science, Chalmers University of Technology, SE-412-96 Gothenburg, Sweden; 2 Department of General Surgery, The General Hospital of Chinese People’s Armed Police Forces, Beijing, China; 3 Non-Communicable Disease Research Unit, South African Medical Research Council and University of Cape Town, Cape Town, South Africa

## Abstract

We investigated the association between dietary patterns and prevalent chronic kidney diseases (CKD), in participants of the 2005–2012 US National Health and Nutrition Examination Survey (NHANES) conducted between 2005 and 2012, who had measured data on dietary intake and kidney function. Analyse of covariance (ANCOVA) and logistic regression models were employed to account for the survey design and sample weights. A total of 21,649 eligible participants (634 with and 20,015 without prevalent CKD) were included in the final analysis. Three food patterns together explained 50.8% of the variance of the dietary nutrients consumption. The first food pattern was representative of a diet containing high levels of saturated and mono-unsaturated fatty acids; the second food pattern comprised vitamins and trace elements; and the third food pattern was mainly representative of polyunsaturated fatty acids. The odd of prevalent CKD decreased across increasing quarters of vitamins and trace elements, so that the top quarter was associated with a 53% (95%CI: 42–62%) lower odds of CKD in age, sex and race adjusted logistic regression models. These results suggest that vitamins and trace elements intake are associated with lower risk of prevalent CKD.

## Introduction

Chronic kidney disease (CKD) is a progressive deterioration of kidney function^1,2^. Cardiovascular disease, anaemia, mineral and bone disorders, peripheral neuropathy and increased infections are important complications of CKD^1,2^. It has been suggested that micronutrient deficiency may contribute to morbidity and mortality in CKD^3^. Diet and lifestyle modifications, and the control of both diabetes and hypertension are important components of strategies to prevent CKD occurrence or slowing the progression. In the field medical nutrition therapy and dietary intervention, few number studies have concluded that dietary Approaches to Stop Hypertension (DASH) and the Mediterranean dietary patterns which are rich in protective nutrients such as antioxidant vitamins including vitamin E, C, A, potassium, magnesium, calcium, fibre, omega fatty acids, and phytochemicals, can affect kidney function and decrease the risk of CKD^1,4^. Studies on general dietary intake in relation with CKD risk have been limited both in numbers and methodologically. Gopinath and co-workers in a sample of 1952 adults older than 50 years, using Healthy Eating Index to determine total diet scores (TDS), reported that compared with Participants in the lowest quarter of TDS, those the highest quarter had a 41% lower risk of having eGFR < 60 ml/min/1.73 m² ^5^. Furthermore, each unit increase in TDS was associated with a 15% lower odds of having prevalent CKD² ^5^. In another study by Gutiérrez and colleagues in 3,972 participants with CKD aged 45 years and above, reported that dietary pattern rich in processed and fried foods was independently associated with mortality. In contrast, a diet rich in fruits and vegetables appeared to be protective^6^.

Considering the potential role of diet in the development and progression of CKD, and limited evidence on the association of dietary food patterns with CKD risk, we used the representative and large sample size in order to investigate the association of dietary food patterns with prevalent CKD in US adults.

## Methods

### Population

The Nutrition and Health Examination Surveys (NHANES) have been extensively described^7,8^. In brief, NHANES are repeated cross-sectional surveys conducted by the National Center for Health Statistics (NCHS). For the current study we used data for participants aged 18 years and above, examined during the 2005–2012 NHANES cycles. In NHANES surveys, data on demographic, dietary, and behavioral information are collected via questionnaires during home visits. Anthropometric measurements and samples collection for biomarker measurements are conducted by trained survey staff at mobile examination units^8–10^. All methods were carried out in accordance with relevant guidelines and regulations^8–10^. All experimental protocols were approved by National Centre for Health Statistics^8–10^. All the participants were aware of study and informed consent was obtained from all subjects^8–10^. Methods for Biochemical analyses are as described in the NHANES Laboratory/Medical Technologists Procedures Manual^8–10^. Creatinine measurement was based on Jaffe reaction and standardized methods^11^. Urinary creatinine by the Jaffe rate reaction, and urinary albumin by solid-phase fluorescent immunoassay, from a random urine sample^12^; were used to calculate the urinary albumin-creatinine ratio (ACR). The CKD Epidemiology Collaboration (CKD-EPI) equation was used to estimate glomerular filtration rate (eGFR, in ml/min/1.73 m²), and eGFR lower than 60 ml/min/1.73 m² used to define prevalent CKD^12^. The National Cholesterol Education Program’s Adult Treatment Panel III report criteria were used to define MetS^13^, based on the presence of 3 or more of the following 5 criteria: (1) waist circumference ≥ 102 cm in men or ≥ 88 cm in women; (2) triglycerides ≥ 150 mg/dL; (3) high-density lipid (HDL) cholesterol < 40 mg/dL in men or <50 mg/dL in women; (4) systolic blood pressure ≥ 130 or diastolic blood pressure ≥ 85 mmHg; (5) fasting blood glucose ≥ 100 mg/dL. The homeostatic model of insulin resistance (HOMA-IR) was calculated using the formula: HOMA-IR = [glucose (nmol/L) * insulin (mU/mL)/22.5], using fasting values^14^.

### Dietary recall

Each food/beverage item and corresponding quantity consumed by each participant from midnight to midnight on the day before the interview was recorded. The in-person dietary recall used a standard set of measurement guides, designed to assist the participant reporting the volume and size of the food/beverage items consumed with accuracy. Following the dietary recall, the energy and nutrient contents of each reported food/beverage item were systematically coded with the U.S. Department of Agriculture’s Food and Nutrient Database for Dietary Studies (FNDDS)^15^.

### Statistical analysis

Data analyses followed the CDC guidelines for analysis of complex NHANES data, accounted for the masked variance and used the recommended weighting methodology, implemented using SPSS complex sample module version 22.0 (IBM Corp, Armonk, NY). Factor analysis with orthogonal transformation (varimax procedure) was applied to derive nutrient patterns based on the nutrients and bioactive compounds. We applied two complementary analytic approaches. In the first approach, we used principle component (PC) factor analysis with Varimax orthogonal transformation to generate PCs representative of dietary patterns based on the highest correlation coefficients between the nutrients constructing each PC^16^. All the necessary prerequisites of PC analysis including linearity, Kaiser–Meyer–Olkin measure of 0.88, and the significant Bartlett’s test of sphericity (p < 0.001) were met. We then used regression methods to calculate the factor scores of each nutrient pattern for each study participants^16^. Factors were retained for further analysis based on their natural interpretation and eigenvalues on the Scree test^17^. We computed the factor score for each nutrient pattern by summing up intakes of nutrients weighted by their factor loadings^18^. Each participant received a factor score for each identified pattern, with increasing score indicating a higher intake of nutrients of target food pattern. Simple linear dose–response relationships are unlikely to be found in nutritional epidemiology^19^. To investigate the association between food pattern an CKD, multivariable (age, sex, race, body mass index, triglyceride, high density lipoprotein, diabetes, and hypertension) regressions were applied. The Nagelkerke pseudo R^2^, was used as global measure of multivariable models performance, and was separately derived for models with covariates only, and for models with covariates and each of the food patterns. The sample-specific distribution of each food pattern variables was used to categorise participants into four groups (quarters) of approximately equal size. This new variable was then used in all regression analyses, with the lowest quarter (first quarter) always used a reference. This approach allowed us to overcome issues relating the departure from normal distribution, of continuous food pattern score, and accordingly, the distortion of regression coefficients from this variable. Multi-collinearity for the multiple linear regressions was assessed with variance inflation factors (VIF) at each step^20^. Multi-collinearity was considered high when the VIF was >10^20^. Averages of cardio-metabolic risk factors levels were compared between participants with and those without CKD. Unless otherwise stated, all tests were two sided, and p < 0.05 used to characterised statistically significant results.

## Results

A total of 40790 participants took part in NHANES across the years under consideration, of whom 21,649 were eligible for inclusion in the current analyses. Of these, 1634 (6.8%) had prevalent CKD. The characteristics of participants overall and by status for prevalent CKD are summarised in Table 1. Overall 11815 (48.9%) participants were men and 12367 (51.1%) were women, with no significant difference by CKD status (p = 0.365). Compared to those without CKD, participants with CKD comprised more non-Hispanic Whites (82.2% vs. 68.4%), and fewer Mexican-Americans (2.7% vs. 8.7%), non-Hispanic Black (8.5% vs. 11.0%), other Hispanic (2.8% vs. 5.2%), or other ethnicities (3.3% vs. 6.7%); p < 0.001 for differences in the distribution of ethnicity by status for CKD. The mean age was 45.9 years overall, and was higher in participants with CKD than in those without (69.0 vs. 44.5 years, p < 0.001). With regard to characteristics by CKD status, people with CKD had higher WC (p < 0.001), higher serum concentrations of hs-CRP, TG and TG/HDL ratio (p < 0.001), fasting and 2-h glucose, insulin, HOMA-IR, and HbA1c (p < 0.001). There were also likely to have high prevalence of MetS, type 2 diabetes mellitus (T2DM), and hypertension (both p < 0.001).

**Table 1:** Demographic characters of subjects based on chronic kidney diseases status.

Characteristics		Overall	With CKD (n = 1634)	Without CKD(n = 20015)	p-value
Sex	Men (%)	48.9	37.7	49.2	<0.001
	Women (%)	51.1	62.3	50.8	
Age (Years), [mean(95% CI)]		45.9 (45.2–46.3)	69.0 (67.9–70.0)	44.5 (43.9–45.1)	<0.001
Race/Ethnicity	White (non-Hispanic) (%)	68.5	82.6	68.4	<0.001
	Non-Hispanic Black (%)	11.6	8.5	11.0	
	Mexican-American (%)	8.3	2.7	8.7	
	Other Hispanic (%)	5.0	2.8	5.2	
	Other (%)	6.6	3.3	6.7	
Body mass index (kg/m^2^)		28.51 ± 0.10	29.45 ± 0.14	28.48 ± 0.10	<0.001
Waist circumference (cm)		97.62 ± 0.26	102.40 ± 0.34	97.48 ± 0.28	<0.001
Serum Triglycerides (mg/dl)		154.22 ± 2.95	177.16 ± 3.60	152.56 ± 1.61	<0.001
Serum Total cholesterol (mg/dl)		195.83 ± 1.28	192.80 ± 1.97	196.06 ± 0.49	0.125
Serum High density lipoprotein (mg/dl)		53.14 ± 0.62	53.00 ± 0.56	53.11 ± 0.22	0.362
Serum triglycerides/HDL cholesterol ratio		3.55 ± 0.10	4.02 ± 0.11	3.51 ± 0.05	0.136
Serum Hs-CRP (mg/dl)		0.39 ± 0.05	0.55 ± 0.02	0.38 ± 0.01	<0.001
Serum Apolipoprotein (B) (mg/dL)		93.24 ± 1.13	92.56 ± 1.49	93.30 ± 0.54	0.532
Systolic blood pressure (mmHg)		121.55 ± 0.56	132.63 ± 0.70	120.62 ± 0.24	<0.001
Diastolic blood pressure (mmHg)		70.16 ± 0.63	70.49 ± 0.25	65.54 ± 0.54	<0.001
Fasting blood glucose (mg/dl)		98.27 ± 0.12	112.45 ± 1.40	97.27 ± 0.34	<0.001
Plasma Insulin (uU/mL)		12.92 ± 0.15	14.59 ± 0.77	12.85 ± 0.17	<0.001
HOMA-IR		3.379 ± 0.08	4.48 ± 0.30	3.30 ± 0.06	<0.001
HbA1c (%)		5.56 ± 0.01	5.99 ± 0.02	5.53 ± 0.01	<0.001
2-hour blood glucose (mg/dL)		115.26 ± 0.65	146.69 ± 3.84	114.24 ± 0.79	<0.001
Hypertension (%)		15.4	34.8	13.9	<0.001
Diabetes (%)		8.6	21.1	7.7	<0.001
Metabolic syndrome (%)		27.1	49.3	27.3	<0.001
First food pattern [Saturated-MUFA](%)					
Q1		21.8	27.3	21.1	<0.001
Q2		23.8	30.5	23.3	
Q3		26.2	23.3	26.4	
Q4		28.3	18.9	29.2	
Second food pattern [minerals and vitamins] (%)					
Q1		23.0	30.3	22.1	<0.001
Q2		24.2	24.0	24.1	
Q3		25.6	26.3	25.7	
Q4		27.3	19.6	28.1	
Third food pattern [Cholesterol-PUFA] (%)					
Q1		24.8	34.2	24.1	<0.001
Q2		24.7	27.0	24.6	
Q3		25.6	23.6	25.8	
Q4		24.9	15.2	25.6	

CKD, chronic kidney diseases; HDL, high density lipoprotein; HOMA-IR, homeostatic model assessment of insulin resistance; hs-CRP, high sensitivity c-reactive protein; MUFA, mono-unsaturated fatty acid; PUFA, polyunsaturated fatty acid; values are expressed as a mean and standard error of the mean; Q1-Q4; quarters. Quarters were derived based on the distribution of each targeted food pattern generating four groups with approximately equal number of participants.

Using a PCA method, we reduced the dietary variables from 63 variables to 3 nutrient patterns that together explained 50.8% of the variance of dietary nutrients consumption. Supplementary Table 1 illustrates the main nutrients that contributed to each nutrient pattern. The first nutrient pattern is mainly representative of saturated fatty acids, mono-unsaturated fatty acids (MUFA), total fat and carbohydrate, the second nutrient pattern represents vitamins, minerals and dietary fiber, and the third nutrient patterns is mainly representative of cholesterol and polyunsaturated fatty acids (PUFA) and protein. Supplementary Table 1 also shows the age, gender, and race adjusted mean of main nutrients by quarters of the three nutrient patterns. For the nutrients that are constituent elements of a nutrient patterns, there are highly statistically significant increases in trends of the nutrient intake by quarters of the corresponding nutrient pattern as expected (p < 0.001). For the nutrients that are not constituent elements of the other nutrient patterns, the trends are less pronounced and non-significant.

The distribution of CKD and non-CKD participants across quarters of food patterns is shown in Table 1. The proportion of participants with prevalent CKD decreased while that for participants without CKD increased or changed less across increasing quarters of the three food patterns. However, after adjustment in logistic regression models, significantly decreasing odds of prevalent CKD was observed only across the quarters of the second food pattern (representing vitamins and trace elements), Table 2. In extended multivariable model, relative to the first quarter, the odd ratio of prevalence CKD was 0.66 (95% CI: 0.54–0.81) for the second quarter, 0.66 (0.54–0.82) for the third quarter and 0.50 (0.40–0.63) for the fourth quarter (Table 2). For the first and third food pattern, while point estimates of the odd ratio of CKD comparing the upper to the first quarter were mostly below unity and therefore suggesting low risk, the confidence around these estimates always included the unity, supporting the non-significance of the estimates. The patterns of the association were similar in minimally adjusted models, that is containing only age, sex and race; or only these augmented with hypertension, triglycerides and high density lipoprotein (Table 2). The Nagelkerke pseudo R^2^ value are shown in Table 2, always reflecting improvement in model performance from adding each of the food patterns to models with covariates only.

**Table 2:** Adjusted logistic regression to examine the association between quartile for food pattern and risk of the chronic kidney diseases.

Food Patterns	Likelihood of CKD with different models					
	Age-Sex-Race		Age-Sex-Race -HTN- TG- HDL		Age-Sex-Race -HTN-DM-TG-BMI-HDL	
	Odds Ratio	Lower Bound-Upper Bound	Odds Ratio	Lower Bound-Upper Bound	Odds Ratio	Lower Bound-Upper Bound
First Food Pattern [Saturated-MUFA] Q2	1.05	0.82–1.35	1.02	0.81–1.40	1.05	0.82–1.35
First Food Pattern [Saturated-MUFA] Q3	0.82	0.65–1.03	0.81	0.64–1.09	0.80	0.64–1.01
First Food Pattern [Saturated-MUFA] Q4	0.82	0.66–1.02	0.80	0.63–1.03	0.83	0.66–1.03
Second Food Pattern [minerals and vitamins] Q2	0.64	0.53–0.77	0.64	0.52–0.79	0.65	0.53–0.80
Second Food Pattern [minerals and vitamins] Q3	0.65	0.54–0.79	0.64	0.56–0.76	0.66	0.53–0.81
Second Food Pattern [minerals and vitamins] Q4	0.47	0.38–0.58	0.49	0.41–0.59	0.50	0.40–0.62
Third Food Pattern [Cholesterol-PUFA] Q2	0.84	0.72–0.98	0.89	0.73–1.10	0.85	0.72–1.00
Third Food Pattern [Cholesterol-PUFA] Q3	0.94	0.78–1.13	0.81	0.70–1.26	0.96	0.79–1.18
Third Food Pattern [Cholesterol-PUFA] Q4	0.82	0.65–1.05	0.79	0.56–1.09	0.85	0.67–1.00
Pseudo R^2^ for model with covariates only	0.327		0.336		0.340	
Pseudo R^2^ for model with covariates and the first food pattern	0.330		0.340		0.344	
Pseudo R^2^ for model with covariates and the second food pattern	0.329		0.339		0.342	
Pseudo R^2^ for model with covariates and the Third food pattern	0.328		0.337		0.341	

The first quarter was used as a reference for all the food patterns. BMI: body mass index, CKD: chronic kidney diseases, DM: Diabetes, HDL: High density lipoprotein, HTN: Hypertension, Q2: Second quarter, Q3: Third quarter, Q4: Fourth quarter, TG: Triglyceride. The pseudo R^2^ is the Nagelkerke pseudo R^2^. Quarters were derived based on the distribution of each targeted food pattern, generating four groups with approximately equal number of participants.

In age-, sex-, race-, fasting blood glucose-, systolic and diastolic blood pressure-, body mass index-, diabetes- and hypertension-adjusted linear regressions, the second food pattern was negatively associated with ACR (β coefficient: −0.033, p < 0.001), while the first and third food patterns were not. All the three food patterns were positively and significantly associated with eGFR [first food pattern, β coefficient: 0.062, p < 0.001; second food pattern, β coefficient: 0.032, p < 0.001; third food pattern, β coefficient: 0.069, p < 0.001]. None of the food patterns was associated with serum creatinine.

## Discussion

We have investigated the associations of dietary food patterns with prevalent CKD and kidney function. The main finding of the present investigation is that a diet rich in vitamins and trace elements is negatively associated with the presence of CKD.

A recent study investigated the status of dietary intake of vitamins in patients with CKD^21^, and reported a negative association between vitamin intake and CK occurence. The Tehran Lipid and Glucose Study^1^ reported that individuals with higher intake of folate, cobalamin, vitamin C, Vitamin D, vitamin E, potassium and magnesium had a reduced risk of CKD, whereas individuals with high intake of sodium had increased risk of CKD^22^. There are conflicting findings on the relationship of dietary minerals intake with CKD risk. An Australian study reported that the intake of magnesium and folate based on estimated average recommended, decreased the risk of CKD by 40–45%^23^. Moreover this study findings suggested a possible role of phosphorus and calcium intake CKD occurrence, which is in contrast with findings of Iranian studies^1^.

Several investigations have proposed potential mechanisms by which dietary micronutrients can reduce the risk of CKD, including via the effects on obesity, hypertension and diabetes^1,24,25^. In line with the role of chronic inflammation in the development of CKD^26^, it has been suggested that micronutrients can play a protective role in the risk of CKD by decreasing inflammatory markers including interleukin 6 (IL6), total homocysteine and CRP^1,27^. In addition, a possible preventive role of some micronutrients including folate, B6 and B12 based on their antioxidant features in end stage kidney diseases, has been proposed^28^.

In contrast to our findings, Huang and colleagues reported that high saturated fatty acids and low linoleic acid were strongly associated with the development of CKD as well as inflammation, insulin resistance and metabolic syndrome^29^. In addition, another investigation has supported the preventive role of total plasma PUFAs, linoleic and linolenic acid in development of chronic renal diseases^30^.

This study is the largest on the association of range kidney function indexes with dietary patterns. Participants were a random sample of the general population and therefore the results obtained from nationally representative samples can be extrapolated to the general population. However, the cross-sectional design of the data collection preclude inference about causality. It is also an expectation that in participants with diagnosed CKD, change in diets is part of the strategies to slow the progression of the disease. It is therefore possible that participants with previously diagnosed CKD would have modified their diet, which in turn could affect our findings, should such participants have been in large number.

Possible clinical and public health implications relate to the fact that extensive knowledge from both proximal and upstream determinants and how they interact to affect the risk, is needed to assist successful prevention and control strategies. To this end, our study provide important information on both deleterious and protective effects of clusters of food items on kidney health, and provide additional insight about pathways of progression and developments of CKD amenable to dietary interventions. While our findings reinforce the importance of balanced diet, the disclosed links between some of the nutrients and CKD may represent novel metabolic pathways and basis for further and future research.

In conclusion, our findings suggest that vitamins and trace elements intake are associated with decreased prevalence of CKD. Whether this knowledge can be exploited for CKD prevention purpose has to be investigated in follow-up studies.

## Electronic supplementary material

Supplementary Info 1
