# Supplementary material for: Food Patterns are Associated with Likelihood of CKD in US Adults
Source: Sci Rep. 2018 Jul 16;8:10696. doi: 10.1038/s41598-018-27365-6 (PMC6048067; doi:10.1038/s41598-018-27365-6)
Supplement: Supplementary file 1 — Supplementary Info 1 [file 41598_2018_27365_MOESM1_ESM.doc]

**Food Patterns Are Associated With Likelihood of CKD in US Adults**

**Running Title:** Food Patterns and Chronic Kidney Diseases

**Mohsen Mazidi^1,2£^ , Hong-kai Gao^3£^,**  **Andre Pascal Kengne^4^**

*1- Key State Laboratory of Molecular Developmental Biology, Institute of Genetics and Developmental Biology,*

*Chinese Academy of Sciences, Beijing 100101, China.*

*2- Institute of Genetics and Developmental Biology, International College, the University of Chinese Academy of Science, Beijing 100101, China.*

*3-Department of General Surgery,The General Hospital of Chinese People’s Armed Police Forces, Beijing, China.*

*4-Non-Communicable Disease Research Unit, South African Medical Research Council and University of Cape Town, Cape Town, South Africa.*

| **Supplementary Table 1: Age, gender, and race adjusted mean of nutrient intakes across quarters of principle component scores representative of nutrient patterns** | | | | | | | | | | | | | | | |
| --- | --- | --- | --- | --- | --- | --- | --- | --- | --- | --- | --- | --- | --- | --- | --- |
| Dietary intakes | **First nutrient pattern (Saturated-MUFA)** | | | | **P-value** † | **Second nutrient pattern (minerals and vitamins)** | | | | **P-value** † | **Third nutrient pattern (Cholesterol**-**PUFA*)** | | | | **P-value** † |
|  | **Q1** | **Q2** | **Q3** | **Q4** |  | **Q1** | **Q2** | **Q3** | **Q4** |  | **Q1** | **Q2** | **Q3** | **Q4** |  |
| **Carbohydrate†** | 201.1±1.4 | 236.8±1.5 | 270.2±1.7 | 339.0±2.4 | <0.001 | 190.8±1.4 | 234.6±1.4 | 273.7±1.4 | 348.0±2.3 | <0.001 | 238.0±1.6 | 255.2±1.5 | 265.1±1.8 | 288.8±2.2 | 0.123 |
| **Total Fat (gm)†** | 46.2±0.4 | 64.2±0.4 | 81.3±0.5 | 120.9±0.6 | <0.001 | 71.7±05 | 73.4±0.4 | 77.9±0.5 | 89.7±0.5 | 0.364 | 51.8±0.5 | 66.2±0.4 | 81.1±0.4 | 113.5±0.6 | <0.001 |
| **Total SFA*(gm)†** | 11.7±0.1 | 18.7±0.1 | 26.5±0.1 | 45.3±0.2 | <0.001 | 24.8±0.2 | 24.3±0.1 | 25.3±0.2 | 27.9±0.2 | 0.236 | 20.6±0.2 | 22.9±0.2 | 25.8±0.1 | 32.9±0.2 | 0.142 |
| **Total MUFA (gm)†** | 17.4±0.2 | 24.1±0.2 | 29.8±0.2 | 42.9±0.3 | <0.001 | 26.2±0.2 | 26.8±0.2 | 28.4±0.2 | 32.8±0.2 | 0.082 | 17.6±0.2 | 23.7±0.2 | 29.8±0.2 | 43.1±0.3 | <0.001 |
| **Dietary Fiber (gm)†** | 15.2±0.2 | 15.3±0.2 | 16.2±0.2 | 19.1±0.2 | 0.065 | 9.4±0.1 | 13.4±0.1 | 17.5±0.1 | 25.6±0.2 | <0.001 | 14.0±0.2 | 15.9±0.2 | 17.0±0.1 | 18.8±0.2 | 0.152 |
| **Protein (gm)†** | 65.8±0.5 | 73.6±0.4 | 81.7±0.4 | 106.4±0.6 | 0.059 | 65.0±0.4 | 74.1±0.4 | 84.1±0.5 | 104.3±0.5 | 0.092 | 57.9±0.4 | 71.7±0.4 | 84.5±0.4 | 113.4±0.6 | 0.026 |
| **Total PUFA (gm)†** | 12.8±0.2 | 15.8±0.1 | 18.0±0.2 | 22.6±0.2 | 0.325 | 14.5±0.2 | 16.0±0.1 | 17.5±0.2 | 21.3±0.2 | 0.253 | 9.0±0.1 | 13.8±0.1 | 18.5±0.2 | 27.9±0.2 | <0.001 |
| **Cholesterol (mg)†** | 231.8±3.2 | 258.5±3.0 | 293.9±2.8 | 411.5±3.9 | 0.082 | 292.8±3.6 | 285.7±3.5 | 289.6±2.6 | 309.6±3.6 | 0.632 | 162.6±2.3 | 223.7±2.5 | 301.5±2.8 | 489.9±5.0 | <0.001 |
| *PUFA: Poly Unsaturated fatty Acid, MUFA: Mono unsaturated fatty acid, SFA: Saturated fatty acid. The highlights indicate the significant contribution of the nutrient as constituent element of the corresponding PC. †P-value for trend of mean nutrient by quarter (Q) of corresponding PC<0.001. | | | | | | | | | | | | | | | |
